# Special issue on teaching social and communicative competences – status quo

**DOI:** 10.3205/zma001468

**Published:** 2021-03-15

**Authors:** Linn Hempel, Rolf Kienle, Claudia Kiessling, Henriette Löffler-Stastka, Swetlana Philipp, Katrin Rockenbauch, Kai P. Schnabel, Anja Zimmermann

**Affiliations:** 1 Brandenburg Medical School Theodor Fontane, Department of Curriculum and Teaching Affairs, Neuruppin, Germany; 2 Charité – Universitätsmedizin Berlin, corporate member of Freie Universität Berlin, Humboldt-Universität zu Berlin, and Berlin Institute of Health, Office of the Vice Dean for Teaching and Learning, Berlin, Germany; 3 Universität Witten/Herdecke, Fakultät für Gesundheit, Lehrstuhl für die Ausbildung personaler und interpersonaler Kompetenzen im Gesundheitswesen, Witten, Germany; 4 Medical University of Vienna, Department of Psychoanalysis and Psychotherapy and Teaching Center/Unit for Postgraduate Programs, Vienna, Austria; 5 University of Jena, Institute of Psychosocial Medicine, Psychotherapy and Psychooncology, Jena University Hospital, Jena, Germany; 6 University of Leipzig, Prorectorate for education and international affairs, Project: "Teaching Practice in Transfer plus" , Leipzig, Germany; 7 University of Bern, Institute for Medical Education, Bern, Switzerland; 8 University of Leipzig, Medical Faculty, Skills- und Simulation Centre LernKlinik Leipzig, Leipzig, Germany

## Introduction

15 years after the foundation of the GMA Committee for Communicative and Social Competences (KusK) and the Basel Consensus Statement [3], a special issue is now available to assess the current situation: How is the landscape of (longitudinal) curricula and courses or examinations of communicative and social competences in the health professions at different locations? After the call for articles, we received a variety of papers that shed light on very different aspects of the training of communicative and social competences. It is particularly pleasing that, in addition to papers from different health professions, articles on different aspects of teaching can also be presented by international teams and thus, in the best case, a broader view may be achieved.

A total of 23 articles are published with this issue. Six of them are research papers, 13 are project reports, one is a short project, two are reviews and one is a commentary. The training and study programs described are pleasingly diverse; they concern students and trainees of midwifery, nursing, physiotherapy and occupational therapy, health promotion and prevention, speech therapy, pharmacy, interpreting students as well as students of human, dental and veterinary medicine. The special issue thus represents a contribution to evidence-based education in the health professions.

The 23 publications submitted have been assigned to six headline topics. For example, some sites report on the challenges of jointly developing and implementing new communication curricula. Two articles in which the authors test methods for teaching communicative competences in order to decide on their permanent implementation in the curriculum conclude the topic of the implementation phase. Four longitudinal curricula anchored in faculties are presented, as well as concrete procedures in individual courses. The topics of interprofessional and online-based communication are also examined in more detail. Last but not least, the topic of assessing communicative competences is addressed. 

The articles that present a curriculum or a course are accompanied by a profile of the status of communicative curricula at the respective faculty, so that one can get a brief and concise overview of the very colorful and diverse landscape of communicative curricula at the various faculties.

This special issue opens with a commentary by Fabry and Kiessling on the question of what communicative competence is and how it can be taught. The commentary aims to be a pragmatic guide for teachers.

## Curriculum development and implementation

Pruskil et al. [4] pointed out the importance of methods of organizational and personal development in the implementation of curricula of social and communicative competences. According to the motto “alone you go fast, together you go far”, Kleinsorgen and colleagues show for veterinary medicine in the SOFTVETS project how development in an international team (Germany, Croatia, Austria, Slovenia, Hungary) can succeed. 

Three further articles are dedicated to the conditions at faculties in the implementation of communicative curricula. Hollinderbäumer and colleagues describe facilitating and hindering factors at the Mainz location and use, for example, a SWOT analysis, while Hinding and colleagues use a case analysis to highlight on the implementation of a standardized communication curriculum at four faculties (Hamburg, Heidelberg, Mainz, and Magdeburg). Pohl and colleagues present an inventory of the communicative skills that are imparted in veterinary medicine at five faculties (Berlin, Hanover, Leipzig, Giessen, Munich). 

## Implementation studies

In order to examine the evidence of communication training, two exciting studies on different teaching methods were conducted. Herchenröther and colleagues from Würzburg piloted a randomised controlled study to argue for the differentiation of didactic methods depending on when they are used during the course of studies, while Pless and colleagues from Bern conducted an implementation study to draw attention to the benefits of video annotations in self-reflective or peer feedback.

## Communication curricula and specific courses

The establishment of longitudinal communication curricula has already been successfully implemented at some locations for several years. Initial routines have been adjusted, challenges have already been analyzed and the solutions integrated. Four locations present their curricula – some of which have already been revised. At three of these locations, teaching has already been established in at least 7 out of 10 semesters.

In Berlin at the Charité, communication has been taught comprehensively since 1999. Freytag, Kienle and colleagues report on planning, integration, teaching and assessment. The challenges involved are also outlined, including the transition from a reformed curriculum in parallel to a regular curriculum to an integrated model program. From Witten/Herdecke, Kiessling and colleagues report on the introduction of a reformed program for medical education called model program medicine 2018+. Since 2018, new thematic focuses have been established, and the longitudinal teaching of communicative competences has been integrated into the focus on “professional and personal development”. In Leipzig, the longitudinal curriculum for communicative competences has become an integral part of the (human) medical curriculum since 2016/17. Zimmermann and colleagues present the origins, the overall concept, the contents and the planned goals for the near future.

The catalogue of competence levels in Austria clearly states the importance of teaching and training to promote effective communication. Exenberger and colleagues provide an overview of the longitudinal communication curriculum at the Medical University of Innsbruck. The presentation of three courses and the presentation of curricular mapping as well as evaluations by lecturers about the importance of communicative competences provide an interesting insight into the development of the curriculum. 

Building a longitudinal communication curriculum requires enormous resources, as requirements have to be met on several levels. Regular quality assurance measures and time are crucial factors in order to be able to maintain a consistent level of the outcome. In addition, a gradual approach to implementation is advisable. Aspects such as quantity, voluntariness and implementation of courses are vividly illustrated by Roller and Eberhard from dentistry in Heidelberg.

Professional communication in the field of veterinary medicine is subject to high complexity in everyday professional life. A teaching project at the University of Hanover is presented by Trittmacher and colleagues, in which animal and environmental observation and the subsequent communication in farm animal husbandry are addressed. The focus of the teaching unit is on identifying and solving difficult discussion situations as well as a concluding reflection statement.

## Distance learning

Face-to-face communication trainings are effective and can convey essential effective elements of health professional communication [2]. In contrast, there is little evidence for communication or communication training via electronic media. The COVID-19 pandemic called for a rapid rethinking and will potentially have far-reaching implications for the teaching and learning of communication elements as well. Even before the pandemic, Ertl, Steinmair and Löffler-Stastka developed a case-based teaching format in Vienna. The aim of the approach was to train interdisciplinary communication, e.g. consultation work and ordering of findings, as well as to consolidate diagnostically relevant “clinical reasoning” processes for good clinical decision-making, in a pure distance mode. Another form of distance learning was evaluated in Bern by Brem and colleagues using an instruction format for telephone emergency communication, which prepares 5^th^ year students for the treatment of medical emergencies. The review based on the student debriefing shows interesting opportunities for improving communication skills. 

## Interprofessional communication

Increasingly complex health care needs require more cooperation and communication between the professional groups involved [5]. Different qualification paths, which are often characterized as silo-like, make it difficult to acquire interprofessional competences during training or during studies [1]. 

Four articles provide examples of how these difficulties can be overcome and how students from different professional groups can learn interprofessionally, i.e. from, about and with each other, during their training or studies: Spiegel-Steinmann and colleagues present the Winterthur conceptional design and implementation of an interprofessional training concept (WIPAKO), which covers five degree programs. The contribution on the topic by Posenau and Handgraaf presents a framework for interprofessional case conferences developed at the University of Applied Sciences in Bochum. This framework is suitable for learning communicative competences in the context of interprofessional case conferences as well as for testing them. Two further articles are dedicated to the connections between specific, interprofessionally conducted courses and changes in certain competencies or attitudes: For example, Mette and Hänze examined the effects of interprofessional peer tutorials on changes in professional stereotypes at the University Medical Centre Mannheim; Strelow and colleagues describe changes in intercultural competence in the context of the PinKo course in Mainz.

## Assessment methods

In addition to interpersonal feedback, self-reflective learning is used as another method for learning and teaching assessment. Gärtner, Prediger and Harendza present the self-assessment method ComCare for communication and interpersonal skills and compared self-reflection with the assessment of simulation patients in Hamburg. In Marburg, Urff and colleagues investigated the validity of an assessment instrument to measure the skill of empathy and aimed to further develop questionnaires along the lines of the Calgary-Cambridge Guide to enable a reliable and objective assessment of communicative skills. Ludwig and colleagues question whether the OSCE format is the supreme discipline among assessment methods for communicative skills. They present the development and validation of a video-based communication-related e-examination in Mainz, which could make resource-saving testing possible. Another innovative assessment method by Selgert and colleagues from Heidelberg aims at the ability to communicate in a patient-friendly way. Here, the written communication of the advanced student with the patient is evaluated.

## Conclusion

The overwhelming amount of diverse work available with this thematic issue shows – 15 years after the first KusK workshop in Basel – the gratifyingly growing community in this field. Communicative and social competencies have arrived and are anchored in the curricula of the medical faculties. They serve to sharpen the profile of the faculties, which in the meantime are not only following a given mandate, but are self-confidently setting priorities. Communicative and social competences are not only taught, but the assessment of social and communicative competencies is met as a challenge. Different formats, various media as well as simulations are used here in a target-oriented way.

Beyond human medicine, colleagues from dentistry, veterinary medicine and the health sciences are also working on the topic, which is also a pleasing development in the last 15 years. Interprofessional approaches also promise new developments that will gain momentum in the coming years. It is now impossible to imagine health profession education without integrated social and communication skills training. The development of digital formats, which has received a significant boost under the current COVID-19 pandemic, should be monitored and carefully examined: Where can digital formats be retained, where can they be used additively? How do they perhaps even more realistically represent the future communication formats in healthcare? Where is nevertheless a lack of relevant elements and how can these be compensated even better for teaching in the future? How helpful are they for learning social and communicative skills in a constantly changing environment?

The present work provides an exemplary insight into various working methods that can be the basis and source of ideas on how the curricular anchoring of social and communicative competencies can succeed, which building blocks appear to be promising, which aspects should be considered when testing and under which conditions new things can be developed. In this special issue, a broad variety of faculties and institutions present their own ideas and approaches from their respective perspectives. These can thus serve as inspiration or reflection on their own work for other contributors to good communication and social skills. We look forward to future academic exchange on this!

## Acknowledgement

We would like to thank all submitters for their work, all reviewers for their valuable support, without whose participation such a special issue would not be possible. We would like to thank Beate Hespelein for her patient and tireless coordination and for her constant and competent addressability.

## Competing interests

The authors declare that they have no competing interests. 

## References

[1] Behrend R, Mette M, Partecke M, Reichel K, Wershofen B (2019). Heterogeneous learning cultures in interprofessional education: a teacher training. GMS J Med Educ.

[2] Datz F, Wong G, Löffler-Stastka H (2019). Interpretation and Working through Contemptuous Facial Micro-Expressions Benefits the Patient-Therapist Relationship. Int J Environ Res Public Health.

[3] Kiessling C, Dieterich A, Fabry G, Hölzer H, Langewitz W, Mühlinghaus I, Pruskil S, Scheffer S, Schubert S (2008). Basler Consensus Statement "Kommunikative und soziale Kompetenzen im Medizinstudium": Ein Positionspapier des GMA-Ausschusses Kommunikative und soziale Kompetenzen. GMS Z Med Ausbild.

[4] Pruskil S, Deis N, Druener S, Kiessling C, Philipp S, Rockenbauch, K (2015). Implementierung von "kommunikativen und sozialen Kompetenzen" im Medizinstudium. Zur Bedeutung von Curriculums-, Organisations- und Personalentwicklung. GMS Z Med Ausbild.

[5] Sottas B, Müller-Mielitz S, Sottas B, Schachtrupp A (2016). "Interprofessionelle Teams sind effizienter und senken die Kosten" - Zur Evidenzlage bei einem kontroversen Innovationsthema. Innovationen in der Gesundheitswirtschaft.

